# Microbial Biofilms and Breast Tissue Expanders

**DOI:** 10.1155/2013/254940

**Published:** 2013-07-16

**Authors:** Melissa J. Karau, Kerryl E. Greenwood-Quaintance, Suzannah M. Schmidt, Nho V. Tran, Phyllis A. Convery, Steven R. Jacobson, Uldis Bite, Ricky P. Clay, Paul M. Petty, Craig H. Johnson, Jayawant Mandrekar, Robin Patel

**Affiliations:** ^1^Division of Clinical Microbiology, Department of Laboratory Medicine and Pathology, Mayo Clinic College of Medicine, 200 First Street S.W., Rochester, MN 55905, USA; ^2^Division of Plastic Surgery, Department of Surgery, Mayo Clinic College of Medicine, Rochester, MN 55905, USA; ^3^Biomedical Statistics and Informatics, Department of Health Sciences Research, Mayo Clinic College of Medicine, Rochester, MN 55905, USA; ^4^Division of Infectious Diseases, Department of Medicine, Mayo Clinic College of Medicine, Rochester, MN 55905, USA

## Abstract

We previously developed and validated a vortexing-sonication technique for detection of biofilm bacteria on the surface of explanted prosthetic joints. Herein, we evaluated this technique for diagnosis of infected breast tissue expanders and used it to assess colonization of breast tissue expanders. From April 2008 to December 2011, we studied 328 breast tissue expanders at Mayo Clinic, Rochester, MN, USA. Of seven clinically infected breast tissue expanders, six (85.7%) had positive cultures, one of which grew *Propionibacterium* species. Fifty-two of 321 breast tissue expanders (16.2%, 95% CI, 12.3–20.7%) without clinical evidence of infection also had positive cultures, 45 growing *Propionibacterium* species and ten coagulase-negative staphylococci. While vortexing-sonication can detect clinically infected breast tissue expanders, 16 percent of breast tissue expanders appear to be asymptomatically colonized with normal skin flora, most commonly, *Propionibacterium* species.

## 1. Introduction

In 2011, 96,277 breast reconstructions using breast tissue expanders were performed in the United States of America by American Society of Plastic Surgeons members [1]. Breast tissue expanders expand skin overlying a mastectomy site, facilitating subsequent implantation of a breast implant.

*Propionibacterium* species are part of the normal flora of the skin, generally considered nonpathogenic, commensal anaerobic bacteria in sebaceous glands of the skin, although clinical infections can occur [2]. *Propionibacterium acnes* and *Staphylococcus epidermidis*, another skin commensal organism, are endogenous breast flora, even in tissue located deep in the gland away from the nipple [3, 4], as a result of their accessing deep breast tissue via the central and intralobular ductal system [3]. Mastectomy surgery anatomically disturbs mammary ducts. Expanders are placed into the breast pocket, usually submuscularly, and gradually expanded over time using interval saline injections administered via a subcutaneous port. The injection port is located close to the shoulder and axillary regions, the normal skin and sebaceous gland flora of which includes *P. acnes* and *S. epidermidis* [2, 5], which are found in higher concentrations on the skin in these regions than on other areas of the body, such as hips or knees [2].

*S. epidermidis *and *P. acnes *have been associated with capsular contracture of breast implants. In a pilot study performed by our group using vortexing-sonication of breast implants, 33% (9/27) of implants removed due to capsular contracture had significant bacterial growth compared to 5% (1/18) of removed implants without capsular contracture (*P* = 0.034) [6]. *Propionibacterium *species and coagulase-negative staphylococci were the most common organisms found. Rieger et al. recently published a larger study using vortexing-sonication and addressing the same issue; breast implant cultures were positive in 45% (40/89), with positive results (most commonly *P. acnes *and coagulase-negative staphylococci) correlating with the degree of capsular contracture [7].

Few studies have addressed the microbiology of breast tissue expanders. Macadam et al. reported bacterial colonization of 43% of 124 expanders but did not use vortexing-sonication [8]. Rieger et al. found that using expander vortexing-sonication, 52% (12/23) of expander cultures were culture positive [7]. 

We previously showed that vortexing-sonication was useful for the detection of biofilm bacteria on the surface of explanted orthopedic metalwork [9–11]. Herein, we evaluated this technique for the diagnosis of infected breast tissue expanders and used it to assess colonization of breast tissue expanders.

## 2. Methods

### 2.1. Study Subjects

We studied consecutive patients who underwent breast tissue expander device removal from April 2008 to December 2011, at the Mayo Clinic in Rochester, MN. Patient characteristics and breast implant-related events were recorded as judged by the treating plastic surgeon. Only participants who had granted permission to have their medical records reviewed for research purposes (Minnesota statute 144.335) were studied. This study was approved by the Institutional Review Board of the Mayo Clinic.

### 2.2. Site Preparation

Prior to surgery, skin was prepared with DuraPrep (3M Health Care, St. Paul, MN, USA) or ChloraPrep with Tint (CareFusion, Leawood, KS, USA). Antimicrobial prophylaxis (typically intravenous cefazolin) was administered one hour prior to surgery. Breast pockets were irrigated with vancomycin (1 g/mL in saline) or DAB solution (1000 mL saline, 20 mg gentamicin and 500,000 units polymyxin B) prior to insertion of the breast tissue expander. For saline expansions performed at Mayo Clinic, the skin was disinfected with 62% ethyl alcohol (Alcare Plus, Steris Corporation, St. Louis, MO, USA) with a second scrub with Povidone-Iodine Swabsticks (Professional Disposables International, Inc., Orangeburg, NY, USA). 

### 2.3. Sample Collection and Processing

For the purposes of the study, the surgeon aseptically placed the removed breast tissue expander into a sterile one- or two-liter polypropylene straight-side wide-mouth jar (Nalgene, Lima, OH, USA). Breast tissue, fluid, and/or a swab were additionally collected using sterile technique on a case-by-case basis as determined by the subject's surgeon. The specimens were sent to the clinical microbiology laboratory for immediate processing in a Class II, Type A2 laminar flow biological safety cabinet. Briefly, 400 mL of Ringer's solution was added, and the container was vortexed for 30 seconds (Vortex Genie; Scientific Industries Inc., Bohemia, NY, USA) and then subjected to sonication (frequency, 40 ± 2 kHz; power density, 0.22 ± 0.04 W/cm^2^) in a Branson ultrasonic bath, model B5510-MT (Branson Ultrasonics, Danbury, CT, USA) for five minutes, followed by additional vortexing for 30 seconds. Fifty milliliters of the resulting sonicate fluid was placed into a sterile conical tube; the tube was centrifuged at 3,150 ×g for five minutes. The supernatant was aspirated, leaving 0.5 mL (100-fold concentration), and 0.1 mL of the sediment was plated onto aerobic and anaerobic sheep blood agar plates, which were incubated aerobically for two to four days and anaerobically for 14 days, respectively. The colony forming units (cfu) per plate (corresponding to cfu/10 mL) were counted, and results were expressed as cfu/10 mL. Results were reported as no growth, <20 cfu/10 mL, 20 to 50 cfu/10 mL, 51 to 100 cfu/10 mL, or >100 cfu/10 mL. Growth of ≥20 cfu/10 mL was considered positive. This cutoff was applied to avoid considering contamination from the patient's skin or contamination during the removal process, transportation, or processing of the expander as a positive result. The cutoff was specifically based on our prior work with prosthetic joints [10, 11]. Prior to culture, tissue specimens were homogenized using a Seward Stomacher Biomaster 80 (Port Saint Lucie, FL, USA) in three mL brain heart infusion broth for one minute. A drop of tissue homogenate was inoculated onto aerobic sheep blood, chocolate, colistin nalidixic acid, and eosin methylene blue agar, and 0.1 mL was placed into thioglycolate broth (BD Diagnostic Systems, Sparks, MD, USA). Fluid cultures were inoculated in the same manner as tissue culture, but thioglycolate broth was inoculated only if anaerobic cultures were ordered. If anaerobic tissue and fluid cultures were ordered, an additional sheep blood and a CDC anaerobic sheep blood agar plate were also inoculated. Aerobic swab cultures were prepared in the same manner but were swished in thioglycolate broth prior to inoculating plates. Aerobic and anaerobic agar plates were incubated at 35 to 37°C in 5 to 7% CO_2_ aerobically for two to four days and anaerobically for seven to 14 days, respectively. Thioglycolate broth was subcultured if turbid. Microorganisms were enumerated and classified using routine microbiologic techniques.

### 2.4. Definitions

Tissue expander infection was defined as the presence of rapidly evolving pain, erythema, fever, local signs of inflammation, intraoperative purulence, and/or surgeon's interpretation. 

### 2.5. Patient Characteristics

For all enrolled patients, the medical records were reviewed to determine age, race, underlying disease(s), history of prior surgeries on the same breast (date and type), results of histopathologic and microbiologic studies, presence or absence of gross purulence (at the time of surgery), breast on which surgery was performed (i.e., left versus right), and reason for surgery (e.g., routine scheduled removal, infection, capsular contracture, rupture of the implant, hematoma or bleeding, chronic pain, extrusion, or leakage of the implant).

### 2.6. Data Analysis

Data were summarized using medians and ranges for continuous variables and percentages for categorical variables. *P* values for continuous variables were from the Wilcoxon rank sum test, and *P* values for categorical variables were from the Fisher's exact test.

## 3. Results

We prospectively evaluated 328 breast tissue expanders removed and collected from 195 surgeries (191 patients) over a 44-month period. Median subject age at the time of explantation was 50 (range, 18–77) years. Median breast tissue expander age from implantation to removal was 10 (range, 1–218) months. Underlying conditions included 55% (179/328) breast cancer, 43% (141/328) prophylaxis, and 2% (8/328) other. All breast tissue expanders had been placed as part of breast reconstruction procedures. Breast tissue expanders were removed for various reason, as follows: 305 were fully expanded, four were removed due to capsular contracture, four due to rupture or leakage, seven due to infection, five to gain symmetry, and three by patient request. One hundred and thirty-three subjects had breast tissue expanders removed simultaneously, and 62 had a single expander removed. Tissue culture was performed in 312 cases; 276 had a single tissue cultured, and only 22 had both aerobic and anaerobic tissue cultures performed. Fluid cultures were performed in 16 cases with both aerobic and anaerobic cultures performed in 13 cases. Additionally, there were eight swabs cultured; all swabs cultures were aerobic.

Seven (2.1%) of the 328 breast tissue expanders were infected, of which six had positive sonicate fluid cultures (Table 1). The single negative sonicate culture had two positive tissue cultures, and the subject's contralateral breast tissue expander was sonicate culture positive. Two infected breast tissue expanders had positive tissue cultures, two had a positive fluid culture (of five tested), and only one had a positive swab culture (of four performed). Median age of the clinically infected subjects was 56 years (range, 35–64 years). Median clinically infected breast tissue expander age was two (range, 1–9) months, lower than nonclinically infected cases (*P* < 0.0001). None of these breast tissue expanders were associated with symptoms of or a history of capsular contracture, and all were first time devices. Following removal of infected breast tissue expanders, new devices were not immediately reinserted. Antimicrobial therapy, irrigation, debridement, and drains were placed in all cases. All patients responded to device removal and antimicrobial therapy. None had recurrent infection. Follow-up records show four of the subjects elected by preference to have no new device inserted, two had silicone gel implants placed, and one was lost to followup.

Of the 328 breast tissue expanders, 321 had no clinical evidence of infection. The median age of these subjects was 50 years (range, 18 to 77 years), and median tissue expander age was 10 months (range, 1–218 months) (Table 2). There were 23 subjects with either a history and/or symptoms of capsular contracture and 176 with breast cancer. In this subpopulation, there were 37 subjects with positive tissue cultures. All subjects had a single tissue submitted for culture except for three subjects who had two tissues samples submitted. Only five had anaerobic cultures performed (see Supplementary Table in Supplementary Material available on at http://dx.doi.org/10.1155/2013/254940).

There were 170 sonicate fluid cultures with growth <20 cfu/10 mL (considered insignificant and not further evaluated).

Among the clinically uninfected subjects, 52 (16.2%, 95% CI, 12.3–20.7%) had positive sonicate fluid cultures (Supplementary Table). Forty-five of the sonicate fluid cultures were positive with *Propionibacterium *species, ten with coagulase-negative staphylococci, two with *Corynebacterium* sp., and one each with *Actinomyces neuii, Pandoraea *species, and *Ralstonia pickettii.* Seven sonicate fluid cultures yielded polymicrobial growth; each had a *Propionibacterium* species present (Supplementary Table). Fourteen clinically uninfected breast tissue expanders with positive sonicate fluid cultures also had growth in tissue culture, 12 with concordant microbiology. The two discordant results grew coagulase-negative staphylococci from two tissue culture samples and *P. acnes* from the sonicate fluid cultures. Median age of the subjects without clinical evidence of infection but with positive sonicate fluid cultures was 45 years (range, 23 to 77 years), and median tissue expander age was 10 months (range, 1–29 months) (Supplementary Table). There were two subjects with either a history of and/or symptoms of capsular contracture and 25 with breast cancer. Of the negative sonicate fluid culture subjects there were 23 single positive tissue cultures (Supplementary Table). Overall, breast tissue expander culture sensitivity was 85.7% (6/7) and specificity was 83.8% (269/321).

There was no evidence that increased subject or breast tissue expander age, textured expander surface, increased number of saline expansions, or radiotherapy was associated with positive sonicate fluid cultures (Table 2).

## 4. Discussion

Results of this study suggest that 16 percent of breast tissue expanders are asymptomatically colonized with normal skin flora, most commonly *Propionibacterium *species, followed by coagulase-negative staphylococci. These organisms are generally considered harmless commensals; however, in the context of foreign bodies, they can be pathogenic. For example, both cause prosthetic joint infection, with *Propionibacterium *species being especially associated with prosthetic shoulder infection [9]. They are also frequently isolated as contaminants, but their isolation in significant quantity from sonicate fluid in this study suggests that they may be colonists of breast tissue expanders, although the clinical significance of this finding remains to be determined. Alternatively, they were isolated as contaminants.

Several factors could theoretically lead to colonization of breast tissue expanders. Bacteria may be introduced through traumatized mammary ducts at time of implantation or during the expansion process. Bacteria have been detected in the saline inside of tissue expanders suggesting that saline expansions may be associated with introduction of bacteria, since the implant envelope is impermeable to bacteria [3, 8].

Our findings are similar to those of numerous other studies on breast tissue, breast milk, and breast implants and their capsules [6, 8, 12–14]. Ransjo et al. sampled breast tissue from 25 patients perioperatively during reduction mammoplasty using an impression pad method and showed a median of 0.1 and 4 cfu/cm^2^ of aerobic bacteria (predominantly *S. epidermidis*) and anaerobic bacteria (predominantly *Propionibacterium *species), respectively, higher than that associated with vascular and orthopedic surgery and with a greater predominance of anaerobes [15]. Thornton et al. isolated coagulase-negative *Staphylococcus *species and *Propionibacterium *species from 53 and 44%, respectively, of 59 breast tissues collected at augmentation or reduction mammoplasty; there was no relationship between the type of bacterium and the depth within tissue where the culture specimens were taken [4].

Coagulase-negative staphylococci and *Propionibacterium* species have been associated with capsular contracture of breast implants. Some suggest that fibrosis is stimulated by biofilms that form on the implant ultimately leading to capsular contracture [6, 7, 16–18]. As discussed in the introduction, prior studies using vortexing-sonication of breast implants suggest an association between positive cultures of breast implants and capsular contracture [6, 7]. Pajkos et al. reported growth from 24/48 (50%) breast implant and/or capsule samples studied; 17/19 samples from patients with capsular contracture were culture positive (mostly for coagulase-negative staphylococci), compared with only 1/8 samples obtained from patients without capsular contracture (*P* = 0.0006) [18]. A link between bacteria and capsular contracture has also been shown in animal studies. A study using miniature gel-filled implants in pigs showed a fourfold increased risk of capsular contracture when implants were inoculated with *S. epidermidis *[19]. A study performed in rabbits with miniature silicone implants showed that implant contamination with *S. epidermidis* resulted in formation of thicker, more fibrotic capsules compared to noncontaminated implants [20]. Whether or not asymptomatically colonized breast tissue expanders are a risk factor for future capsular contracture remains to be determined. Radiotherapy has been implicated in causing/exacerbating capsular contracture as well as increasing the risk of infection [8, 12, 21–24]. In our study, however, there were 56 subjects who underwent radiotherapy, only two of whom had positive sonicate fluid cultures.

The finding of positive sonicate fluid cultures in our study occurred despite preoperative administration of cefazolin and surgical skin preparation as well as preexpansion skin preparation. Saltzman et al. examined skin colonization of shoulder sites and showed that after surgical skin preparation with ChloraPrep, DuraPrep, or povidone-iodine, 7, 12, and 15% of cultures were positive for *P. acnes *and 2, 4, and 19% were positive for coagulase-negative staphylococci, respectively [5]. Preoperative antibiotics may have reduced the recovery of bacteria from tissues and breast tissue expanders leading to falsely negative results. 

Our study has important limitations. Tissue culture was performed in most cases, but only 36 subjects had more than one tissue specimen cultured. For diagnosis of implant-associated infection/colonization, especially with commensal flora, it would have been ideal (for optimal sensitivity and specificity) to culture multiple tissue specimens. There were 23 expanders with positive tissue but negative sonicate fluid (<20 cfu/10 mL) cultures, of which 18 had between 1 and 20 cfu/10 mL of growth from sonicate fluid culture. It is possible that in some cases, bacteria are present in breast tissue but not on the surface of the implant. The significance of the 170 expanders with growth of <20 cfu/10 mL from sonicate fluid cultures is most likely attributable to contamination. While anaerobic cultures were performed on all sonicate fluids, they were only performed on tissues from 22 subjects, limiting assessment of isolation of *P. acnes *by tissue culture. These limitations make it challenging to compare the results of tissue and sonicate fluid cultures. 

Our study shows that while sonication can detect clinically infected breast tissue expanders, 16 percent of breast tissue expanders appear to be asymptomatically colonized with normal skin flora, most commonly, *Propionibacterium *species. Sonication of the device is not warranted in the cases of routine removal of breast tissue expanders but may be useful where infection is evident to identify the pathogen(s). Whether or not the positive sonicate fluid cultures in the clinically uninfected subjects are a risk factor for future infection or capsular contracture remains unknown. Follow-up studies are warranted.

## Supplementary Material

Among subjects with no clinical evidence of infection, there were 37 with positive tissue and 52 with positive sonicate fluid cultures (≥20 cfu/10 ml), 12 of whom had concordant microbiology. Sonicate fluid cultures detected *Propionibacterium* sp. in 45 instances.

## Figures and Tables

**Table 1:** Culture results among subjects with a clinical diagnosis of infection.

Sample-patient ID	Sonicate fluid culture (cfu/10 mL)	Tissue culture (number positive/number taken)	Swab culture (number positive/number taken)	Fluid culture (number positive/number taken)
1-1	20–50 coagulase-negative *Staphylococcus *sp.	Negative (0/3)*	Negative (0/1)**	Negative (0/3)*

85-51	20–50 coagulase-negative *Staphylococcus *sp., >100 *Propionibacterium acnes *	Negative (0/1)**	Not done	Negative (0/1)*

155-93	Negative	Coagulase-negative *Staphylococcus *sp. (2/2)**	Negative (0/1)**	Not done
156-93	20–50 coagulase-negative *Staphylococcus *sp.	Negative (0/3)**	Negative (0/1)**	Not Done

177-106	51–100 *Serratia marcescens *	Negative (0/1)**	*S. marcescens* (1/1)**	*S. marcescens* (1/1)**

196-119	51–100 coagulase-negative *Staphylococcus *sp.	Negative (0/2)*	Not done	Coagulase-negative *Staphylococcus *sp. (1/1)*

309-185	20–50 *Staphylococcus aureus *	*S. aureus* (1/1)**	Not done	Not done

*Aerobic and anaerobic cultures.

**Aerobic culture only.

**Table 2:** Demographics of the subjects with no evidence of infection.

Characteristic	Sonicate fluid culture		*P* value
	Positive (≥20 cfu/10 mL)	Negative (<20 cfu/10 mL)	
Number of breast tissue expanders (number of subjects)	52 (39)	269 (166)	
Subject age (years) (median [range])	45 (23–77)	52 (18–77)	<0.0001
Breast tissue expander age (months) (median [range])	10 (1–29)	11 (2–218)	0.5221
Breast tissue expander surface			1.00
Smooth	1	10	
Textured	47	257	
Saline expansions at Mayo Clinic Rochester (*n* = 209)	33	176	
Total number of expansions (median [range])	7 (1–15)	8 (0–20)	0.0175
Time since first expansion (days) (median [range])	298 (37–1127)	320 (43–2684)	0.2865
Time since last expansion (days) (median [range])	175 (29–1222)	172 (1–2588)	0.364
Number of breasts with radiotherapy treatment	2	54	0.0026
Underlying condition (%)			0.1742
Breast cancer	25 (48.1)	151 (56.1)	
Prophylaxis	23 (44.2)	114 (42.4)	
Other	4 (7.7)	4 (1.5)	
