# Supplementary material for: Microbial Biofilms and Breast Tissue Expanders
Source: Biomed Res Int. 2013 Jul 16;2013:254940. doi: 10.1155/2013/254940 (PMC3730356; doi:10.1155/2013/254940)
Supplement: Supplementary file 1 — Among subjects with no clinical evidence of infection, there were 37 with positive tissue and 52 with positive sonicate fluid cultures (≥20 cfu/10 ml), 12 of whom had concordant microbiology. Sonicate fluid cultures detected Propionibacterium sp. in 45 instances. [file 254940.f1.docx]

Supplementary Table. Culture results among subjects with no indication of infection and positive sonicate culture (≥20 cfu/10 ml) and/or tissue culture.

| **Sample** | **Patient** | **Tissue Culture**  **(number positive/number collected)** | **Sonicate Culture**  **(cfu/10 ml)** |
| --- | --- | --- | --- |
| 152 | 91 | Not done | >100 *Propionibacterium acnes* |
| 13 | 8 | Negative (0/1) | >100 *P. acnes* |
| 15 | 9 | Negative (0/1) | 20-50 *P. acnes* |
| 55 | 33 | Negative (0/1) | >100 *P. acnes* |
| 56 | 34 | Negative (0/1) | 51-100 *P. acnes* |
| 63 | 39 | Negative (0/1) | 51-100 *P. acnes* |
| 65 | 40 | Negative (0/1) | >100 *P. acnes* |
| 66 | 40 | Negative (0/1) | 20-50 *P. acnes*,  51-100 *Pandorea* sp.,  20-50 *Ralstonia pickettii* |
| 72 | 43 | Negative (0/1) | >100 *P. acnes* |
| 95 | 57 | Negative (0/1) | 20-50 *P. acnes* |
| 108 | 65 | Negative (0/1)* | 20-50 Coagulase negative *Staphylococcus* sp. |
| 118 | 71 | Negative (0/1) | 20-50 Coagulase negative *Staphylococcus* sp. |
| 121 | 72 | Negative (0/1) | 20-50 *P. acnes* |
| 124 | 74 | Negative (0/1) | 20-50 *P. acnes* |
| 125 | 74 | Negative (0/1) | 20-50 *P. acnes* |
| 139 | 82 | Negative (0/1) | 20-50 *P. acnes* |
| 140 | 82 | Negative (0/1) | >100 *P. acnes,*  51-100 Coagulase negative *Staphylococcus* sp. |
| 144 | 85 | Negative (0/1) | 51-100 *P. acnes* |
| 145 | 85 | Negative (0/1) | 20-50 *P. acnes* |
| 153 | 92 | Negative (0/1) | 51-100 *P. acnes* |
| 166 | 99 | Negative (0/1) | 51-100 *P. acnes* |
| 174 | 104 | Negative (0/1) | 20-50 *P. acnes* |
| 178 | 107 | Negative (0/1) | 20-50 *Corynebacterium* sp. |
| 187 | 112 | Negative (0/1) | 20-50 *P. acnes* |
| 191 | 115 | Negative (0/1) | 20-50 Coagulase negative *Staphylococcus* sp. |
| 207 | 125 | Negative (0/1) | >100 *P. acnes* |
| 214 | 129 | Negative (0/2) | 51-100 *P. acnes* |
| 215 | 129 | Negative (0/1) | >100 *P. acnes,*  20-50 Coagulase negative *Staphylococcus* sp. |
| 216 | 130 | Negative (0/2) | >100 *P. acnes* |
| 264 | 159 | Negative (0/1) | >100 *Propionibacterium avidum*,  20-50 *Actinomyces neuii* |
| 269 | 162 | Negative (0/1) | 20-50 *P. acnes* |
| 270 | 162 | Negative (0/1) | 20-50 *P. acnes* |
| 276 | 166 | Negative (0/1) | 20-50 *P. acnes* |
| 280 | 168 | Negative (0/1) | >100 *P. acnes* |
| 295 | 176 | Negative (0/1) | >100 *Staphylococcus saccharolyticus* |
| 296 | 176 | Negative (0/1) | >100 *P. acnes*,  20-50 *S. saccharolyticus* |
| 298 | 178 | Negative (0/2) | 20-50 *P. acnes* |
| 320 | 191 | Negative (0/2) | >100 *P. acnes* |
| 64 | 39 | *Propionibacterium* sp. (1/1) | 20-50 *P. acnes* |
| 71 | 43 | *Propionibacterium* sp. (1/1) | 20-50 *P. acnes* |
| 110 | 66 | Coagulase negative *Staphylococcus* sp. (1/1) | 20-50 Coagulase negative *Staphylococcus* sp. |
| 133 | 79 | *P. acnes* (1/1)* | >100 *P. acnes*,  20-50 *Corynebacterium* sp. |
| 134 | 79 | *P. acnes* (1/1)* | >100 *P. acnes* |
| 148 | 88 | Coagulase negative *Staphylococcus* sp. (1/1) | 51-100 Coagulase negative *Staphylococcus* sp. |
| 154 | 92 | *Propionibacterium* sp. (1/1) | 51-100 *P. acnes* |
| 203 | 123 | *Propionibacterium* sp. (1/1) | 51-100 *P. avidum*,  >100 Coagulase negative *Staphylococcus* sp. |
| 213 | 128 | Coagulase negative *Staphylococcus* sp. (2/2) | >100 *P. acnes* |
| 274 | 165 | *Propionibacterium* sp. (1/1) | 20-50 *P. acnes* |
| 275 | 165 | *Propionibacterium* sp. (1/1) | >100 *P. acnes* |
| 293 | 175 | *P. acnes* (1/1) | >100 *P. acnes* |
| 294 | 175 | *Propionibacterium* sp. (1/1) | >100 *P. acnes* |
| 299 | 179 | Coagulase negative *Staphylococcus* sp. (2/2) | 20-50 *P. acnes* |
| 3 | 3 | Coagulase negative *Staphylococcus* sp. (1/1) | Negative |
| 5 | 4 | Coagulase negative *Staphylococcus* sp. (1/1) | Negative |
| 6 | 4 | Coagulase negative *Staphylococcus* sp. (1/1) | Negative |
| 16 | 10 | Coagulase negative *Staphylococcus* sp. (1/1) | Negative |
| 35 | 20 | *Propionibacterium* sp. (1/1) | Negative |
| 43 | 25 | *Propionibacterium* sp. (1/1) | Negative |
| 58 | 35 | *Propionibacterium* sp. (1/1) | Negative |
| 81 | 49 | *Propionibacterium* sp. (1/1) | Negative |
| 88 | 53 | *Propionibacterium* sp. (1/1) | Negative |
| 89 | 53 | *Staphylococcus. aureus* (1/1) | Negative |
| 91 | 55 | Coagulase negative *Staphylococcus* sp. (1/2) | Negative |
| 115 | 69 | Non-fermenting gram negative sp. | Negative |
| 122 | 73 | *Propionibacterium* sp. (1/1) | Negative |
| 123 | 73 | *Propionibacterium* sp. (1/1) | Negative |
| 127 | 76 | Coagulase negative *Staphylococcus* sp. (1/1) | Negative |
| 212 | 128 | Coagulase negative *Staphylococcus* sp. (1/1) | Negative |
| 242 | 145 | Coagulase negative *Staphylococcus* sp. (1/1) | Negative |
| 254 | 151 | Streptococcus viridans gp. (1/1) | Negative |
| 258 | 154 | *Propionibacterium* sp. (1/1) | Negative |
| 282 | 169 | Coagulase negative *Staphylococcus* sp. (1/1) | Negative |
| 300 | 180 | *P. acnes* (1/1)* | Negative |
| 319 | 191 | *Propionibacterium* sp. (1/2) | Negative |
| 326 | 194 | *P. acnes* (1/1)* | Negative |

*Aerobic and anaerobic cultures
